# Fire alters diversity, composition, and structure of dry tropical forests in the Eastern Ghats

**DOI:** 10.1002/ece3.7514

**Published:** 2021-05-01

**Authors:** U. V. Neeraja, S. Rajendrakumar, C. S. Saneesh, Venkat Dyda, Tiffany M. Knight

**Affiliations:** ^1^ Institute of Biology Martin Luther University Halle‐Wittenberg Halle (Saale) Germany; ^2^ German Centre for Integrative Biodiversity Research (iDiv) Halle‐Jena‐Leipzig Leipzig Germany; ^3^ Department of Community Ecology Helmholtz Centre for Environmental Research‐ UFZ Halle (Saale) Germany; ^4^ Centre for Sustainable Future Department of Chemical Engineering and Materials Science Amrita Vishwa Vidyapeetham Coimbatore India; ^5^ Foundation for Ecological Security Madanapalle India

**Keywords:** Eastern ghats, forest fire, species composition, species diversity, tropical dry forest

## Abstract

Fire is known to have dramatic consequences on forest ecosystems around the world and on the livelihoods of forest‐dependent people. While the Eastern Ghats of India have high abundances of fire‐prone dry tropical forests, little is known about how fire influences the diversity, composition, and structure of these communities. Our study aimed to fill this knowledge gap by examining the effects of the presence and the absence of recent fire on tropical dry forest communities within the Kadiri watershed, Eastern Ghats. We sampled plots with and without evidence of recent fire in the Eswaramala Reserve Forest in 2008 and 2018. Our results indicate that even though stem density increases in the recently burned areas, species richness is lower because communities become dominated by a few species with fire resistance and tolerance traits, such as thick bark and clonal sprouting. Further, in the presence of fire, the size structure of these fire‐tolerant species shifts toward smaller‐sized, resprouting individuals. Our results demonstrate that conservation actions are needed to prevent further degradation of forests in this region and the ecosystem services they provide.

## Graphical abstract

Dry tropical forests in the Eastern Ghats have higher density of woody plant stems in recently burned plots, but lower species richness because communities become dominated by a few species with fire resistance and tolerance traits, such as thick bark and clonal sprouting. In the presence of fire, the size structure of these fire‐tolerant species shifts toward smaller‐sized, resprouting individuals.

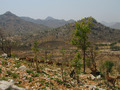

## 1 INTRODUCTION

Species richness and composition are important indicators of forest biodiversity and are known to change across natural and anthropogenic environmental gradients (Barlow et al., 2016; Husch et al., 2002). Fire is an important disturbance factor that alters forest ecosystems around the world (Condé et al., 2019; De Andrade et al., 2020; He et al., 2019). Globally, more than 90% of forest fires are linked to human activity (FAO 2007). Fire is used for agricultural activities, maintaining grasslands for livestock, extracting forest products, cultivation, and hunting (FAO 2007). Annually, fires burn up to 500 million hectares of woodland, open forests, tropical and subtropical savannas, 10–15 million hectares of boreal and temperate forest, and 20–40 million hectares of tropical forests (Bunk, 2004). In recent years, there is an increase in the number of forest fires and burned area in several ecosystems worldwide (Chuvieco et al., 2004; Cochrane, 2003; Dimitrakopoulous et al., 2011; Kodandapani et al., 2004; Westerling, 2016). Tropical dry forests are especially prone to human‐mediated fires because they have a distinct dry season in which dry vegetation such as grasses provides natural fuel for fires (Janzen, 1988a; Murphy & Lugo, 1986).

Fire is known to reduce the abundance and diversity of species in forests (Barlow et al., 2016; Condé et al., 2019; De Andrade et al., 2020), by influencing demographic processes such as survivorship, flowering, seed dispersal, and recruitment (De Luis et al., 2005; Prestes et al., 2020; Verma et al., 2017; Walters et al., 2004). Fire alters species composition, stage structure, and successional patterns through its varying effects across species (Syaufina & Nuruddin, 2011). Nonsprouting woody species with thin bark are especially sensitive to fire (Gosper et al., 2012; Hoffman et al., 2012; Keeley, 1986). The effects of fire on forest communities depend on the frequency and intensity of the fires (Bond & Keeley, 2005; He et al., 2019; Lewis & Debuse, 2012).

The Indian subcontinent is spatially diverse in its climate, forest vegetation types, flammability, and human impact (Champion & Seth, 1968; Ravindranath & Sukumar, 1998). Tropical dry forests account for 40.86% of the total forest cover (FSI, 2019). Fires are most common in this forest type and are increasing in their frequency and spatial extend through time (FSI, 2012; Reddy et al., 2017; Srivastava & Garg, 2013). Many people live in and around dry forest areas and rely largely on forest resources for their livelihood (Kothari et al., 1995; Schmerbeck & Fiener, 2015). Ecosystem services provided by these forests include timber and nontimber forest products, fuelwood, fodder, and places for worship (Sagar et al., 2003; Saha, 2002; Schmerbeck & Fiener, 2015; Singh & Singh, 2011). Fires in India are mainly anthropogenic, caused intentionally (e.g., because resprouting grasses are palatable to livestock) or negligently (Joseph et al., 2009; Roy, 2003; Saha, 2002). Forest fires occur during the dry season from February to June, and these are typically ground fires (Joseph et al., 2009) that spread due to fuel from the accumulation of dry litter, and dry grasses or herbaceous groundcover. Weather conditions during the dry season, such as strong winds, low humidity, and high temperature, also influence burning (Giriraj et al., 2010; Kodandapani et al., 2008). With enough fuel, wildfires can develop that go to treetops. Fires in Indian dry tropical forests have been shown to reduce recruitment, decrease forest diversity, and alter species composition (Chaturvedi et al., 2017; Mondal & Sukumar, 2015; Saha & Howe, 2003; Verma et al., 2017), especially if fires are frequent and intense (Jhariva et al., 2012; Kodandapani et al., 2008; Sathya & Jayakumar 2017; Verma & Jayakumar, 2015). Tree species that can persist have functional traits that allow them to resist or tolerate fire, such as thick bark and resprouting ability (Khan & Tripathi, 1989; Saha & Howe, 2003).

There is limited knowledge on the effects of fire on dry tropical forests in the Eastern Ghats of India. Approximately 14% of forest fires in India occur in Andhra Pradesh (Eastern Ghats; Reddy et al., 2017; Vadrevu, 2006), and there is concern that these fires may have increased in frequency across the past decade in response to changes in livestock practices toward more fodder‐dependent species (FES, 2011; FES social survey 2018 unpublished). Dry tropical forests are the most common habitat type in Andhra Pradesh. An increase in burnt area (from 6,369 km^2^ in 2009 to 8,593.5 km^2^ in 2012) in Andhra Pradesh has been reported, with the Eastern Ghats and Deccan Plateau being the most affected geographical regions within the state (Reddy et al., 2012). The Eastern Ghats are spread across ~75,000 square kilometers covering the states of Orissa, Andhra Pradesh, southern Tamil Nadu and Karnataka, and have high biodiversity and many species that are endemic to the region (Sandhyarani et al., 2007). This region is home to a large human population with high forest dependency. The growth in human and livestock density has increased the demand for forest products (Bahuguna & Singh, 2002; Krishna & Reddy, 2012). However, forests in the Eastern Ghats have received little scientific attention, and as a result, little is known about the effects of fire on these forests. It is important to understand the drivers of tree species abundance, diversity, composition, and structure for conservation and management of biodiversity and to provide local livelihood needs (Davidar et al., 2010; Singh & Singh, 2011).

Our study evaluates (a) whether there has been an increase in the frequency of fires in forests in the state of Andhra Pradesh from 2001 to 2018, (b) tree species diversity and composition in recently burned and not burned plots in the southcentral Eastern Ghats in 2008 and 2018, and (c) stem density and stage structure of common tree species in burned and not burned plots and whether this depends on their fire tolerance traits in 2018. We expect that fire might have increased in its frequency across the past two decades and between our sampling periods of 2008 and 2018. We expect that fire presence has an influence on the density, diversity, composition, and stage structure of tree communities. In particular, we expect that fire‐tolerant species will become more abundant and that these species will be present in smaller stage classes in plots with recent fire presence, indicative of recent resprouting.

## 2 MATERIALS AND METHODS

### 2.1 Study area

**FIGURE 1:**
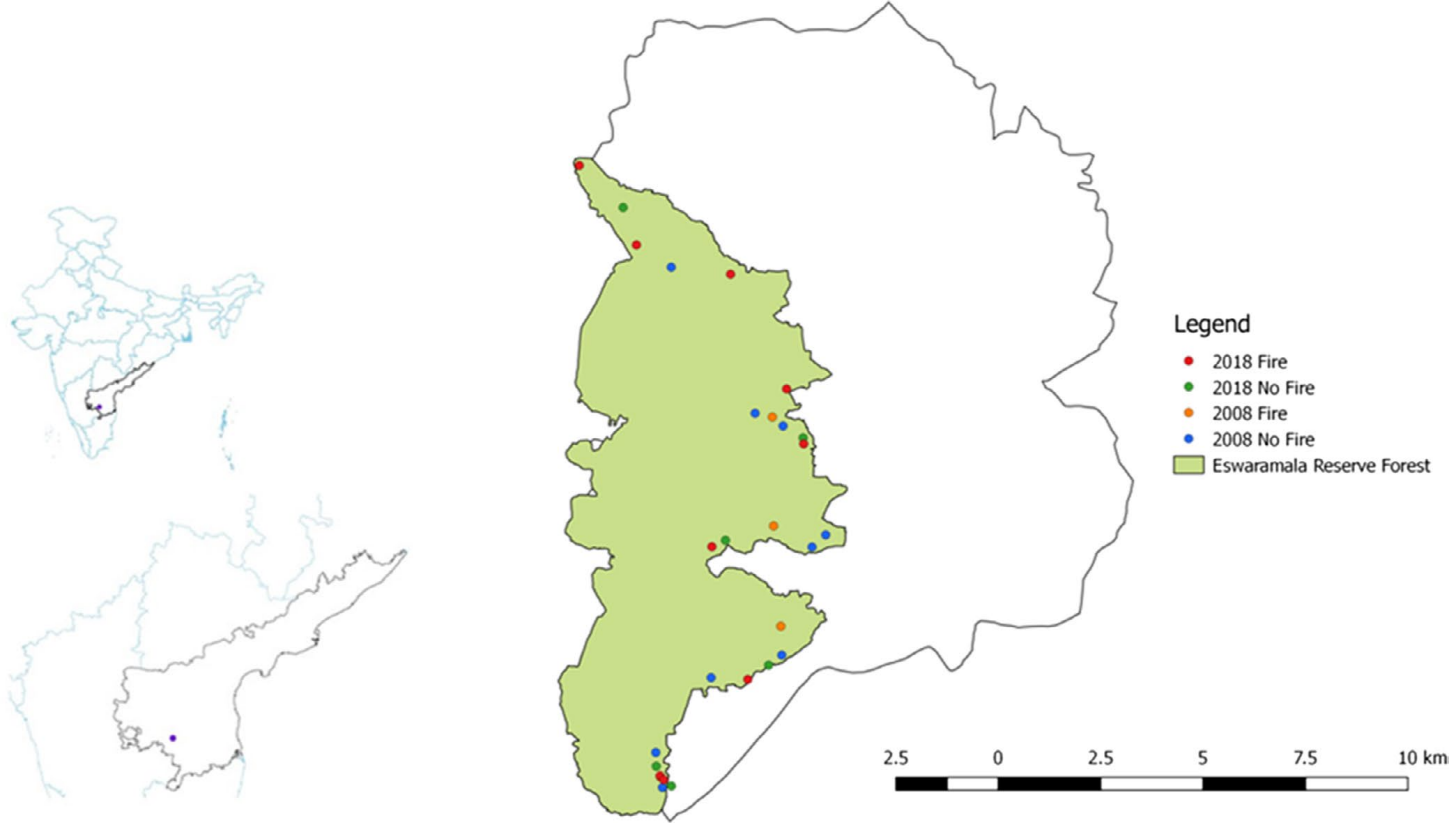
Maps showing the study location. Maps on the left show this study area within the context of the country (India) and state (Andhra Pradesh). The map on the right shows the Kadiri watershed. Our sampling locations (points) are within the Eswaramala Reserve Forest. Sampling locations are color‐coded based on the year of study and whether or not recent fire occurred

Our study area, the Eswaramala Reserve, is located in the Kadiri watershed in the southcentral Eastern Ghats within the Anantapur District, Andhra Pradesh (Figure 1). The Anantapur district is the second driest region in India (Rao et al., 2013), and the Kadiri watershed lies between 13°56'20" and 14°7'20" North and 78°19'30" and 78°27'20" East. The Eastern Ghats are known to have a wide range of topographical and physical features and to harbor rich and diverse flora (Reddy et al., 2008). The main river that drains the Kadiri watershed is Papagni, a tributary of River Pennar. The geographical extent of the watershed is ~185 km^2^. Topography is undulating with elevations ranging from 300m to 1300m. The climate is tropical arid, with a mean annual temperature of 33.7°C and a mean annual rainfall of 553 mm (FES, 2011). The Kadiri watershed falls under the dry rain shadow area of the southern Deccan plateau. The rainfall is unevenly distributed in time, with long periods of drought being common. The soil types are classified as red and black sandy, clay, and red loamy. The main rock types in the study area are peninsular gneisses, younger granites, Dharwar schist belt rocks with secondary intrusives such as dykes, and quartz and pegmatite veins (FES, 2011).

Land use in the Kadiri watershed is classified as forest, agriculture, grassland, and open barren. Forest is the second largest land use and occupies over 62.23 km^2^ (FES, 2011). Our study focuses on forests, which are situated within the Eswaramala Reserve Forest in the western side of the Kadiri watershed. These forests are best described as tropical dry deciduous and tropical thorny–scrub vegetation (Champion & Seth, 1968; Gadgil & Meher‐Homji, 1986). Perennial tall C4 grasses, mainly *Cymbopogon coloratus*, dominate the forest floor (FES, 2011; Rajendrakumar, 2014). The high human density results in high dependency on forests to support rural livelihoods and economy. The forest experiences anthropogenic disturbances, including cutting and lopping of trees for fodder, fuelwood, brick making, timber and nontimber forest products, grazing by sheep and goats, and fires (FES, 2011; FES social survey 2018; Rajendrakumar, 2014). Soil erosion is often a consequence of these anthropogenic disturbances (Krishnaiah, 2013).

In the Eswaramala Reserve, fires occur in the dry season (between February and April) in patches across the forest (FES, 2011). The most common reason for start of fires in this system is burning to promote new flush of grasses for grazing livestock during the dry season. Fires do not seem to be set strategically but are rather set at random locations. Once started, fires spread naturally, the spread is governed by understory grasses (in the form of tall C4 grasses) and the local weather conditions (i.e., wind) on that day. There are natural firebreaks, which can prevent fires from spreading such as regular paths created by grazing livestock and shepherds, rockiness, and contour trenches. Some fires are stopped by forest department or village institution members that are trained to put out fires, to prevent fires from spreading over larger areas. Due to the frequent occurrence of fire, fire protection methods such as fire lines and basal area clearing (0.5m radius) around younger trees are prepared in advance by community members to prevent fires from burning large areas. Fires typically burn standing grass and younger woody plant biomass, and high‐intensity crown fires are less common.

### 2.2 Frequency of fire through time

To test whether the frequency of fire is increasing across time for forests in our region, we examined annual satellite samples of fire presence from 2001 to 2018 in the forested regions within the state of Andhra Pradesh. Data were obtained from the Forest Survey of India Forest fire portal (http://117.239.115.41/smsalerts/index.phpFSI). Detected forest fire points (1‐km^2^ resolution) are from MODIS satellites (Terra and Aqua). These include fresh and repeated detections of active fire at two time points in each day as the satellites pass over the region. From 2001 to 2018, the methods used by MODIS are consistent, allowing a test of whether the frequency of fire increases with time. We tested for a relationship between fire frequency through time by fitting a generalized additive model using the function *gam* from the package “mgcv” in R.

### 2.3 Data collection

**FIGURE 2:**
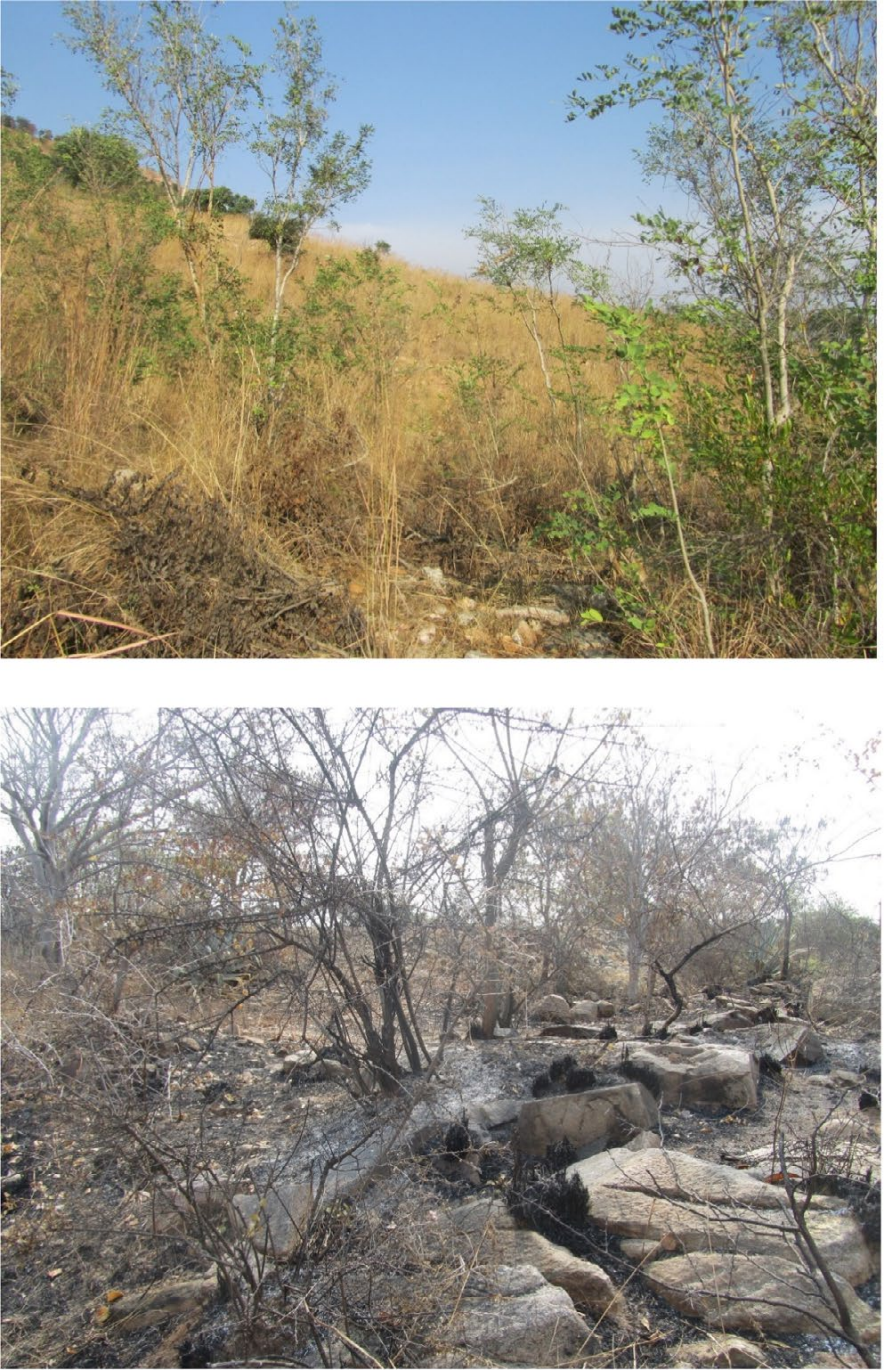
Picture of a plot with no fire (top) and with fire (bottom). In the absence of fire, ground vegetation is present. After a fire, ground vegetation is absent and trees show fire marks

To quantify the richness and composition of trees in 2008 and 2018, we sampled circular plots with a 10 m radius. We focus on tree species (not herbs, shrubs) in this study and define a tree as either a woody plant capable of a height of at least two meters (for single‐stemmed woody plants) or capable of having at least one of the stems with a girth of more than five centimeters (for multistemmed woody plants) (IUCN Global Tree Specialist Group; Beech et al., 2017). In 2008, plots were selected to represent the entire range of topography and disturbance regimes, while allowing for accessibility. In 2018, plots were selected to be in the same approximate locations as the 2008 plots, to capture the same range of topography and disturbance regimes. At each plot, all individual trees with greater than 2‐cm basal girth were identified and counted, including young stage classes, such as saplings and small resprouting individuals. All the individuals were identified using the guide to the regional flora, viz. Flora of Andhra Pradesh vol I, II, and III (Pullaiah, 1997; Pullaiah & Ali Mouali, 1997; Pullaiah & Chennaiah, 1997). We measured girth at breast height (GBH) of stems above 20 cm in 2008, and GBH (or collar girth when GBH was not possible) of all stems in 2018. At each plot, we document the distance to nearest human habitation (distance to village center), and only plots that were at least 500 m away from these were considered in our analysis. In addition, we removed plots that contained less than two individual tree stems. We therefore included 38 plots in 2008 and 48 plots in 2018. We document evidence of recent fires by visual estimation of plots (see pictures in Figure 2). Eight of the 38 plots in 2008 and 29 of the 48 plots in 2018 had evidence of recent fire (i.e., fire marks on trees, lack of ground vegetation).

In 2008, there were 9 sampling locations with 2–6 plots in each location, each plot separated by a minimum of 100 m (Figure 1). In 2018, there were 10 locations, each with 2–8 plots separated by a minimum of 100 m (Figure 1). In both 2008 and 2018, we find that there is a weak relationship between pairwise geographical distance between plots and pairwise community dissimilarity (see Appendix S1A), likely because of both natural (slope, aspect, soil features) and anthropogenic (fire) factor turnovers at small spatial scales. For this reason, we treat each plot as an independent replicate.

### 2.4 Richness and composition

We quantified whether richness and community composition of trees differ between four categories (2008 fire, 2008 no fire, 2018 fire, and 2018 no fire). Richness was assessed using individual‐based rarefaction curves for each category, and their 95% confidence intervals generated from resampling using *iNext* package in R (Chao et al., 2014; Hsieh, 2016). Rarefaction curves with nonoverlapping 95% confidence interval were considered significantly different. We used nonmetric multidimensional scaling (NMDS) to visualize composition between the four categories. We performed NMDS using the *metaMDS* function and the Bray–Curtis dissimilarity matrix in *vegan* package (Oksanen et al., 2015) and used the *ggplot2* package for graphical representation. We used ANOSIM (analysis of similarities) from the *vegan* package to test whether categories differed in their community composition (Bray–Curtis). We removed eight plots from visualizations and analyses that included only one tree species, as two of these plots were clear outliers (Appendix S1B). All statistical analyses and visualizations were conducted in R programming version 3.6.2 (R Core Team, 2017).

### 2.5 Stem density and size structure

We used the more detailed data collection in 2018, for which the size of every stem was measured, to assess how the fire categories influence the density of stems per plot across all individual and the size structure of stems for 10 species with adequate sample size. We used the Kruskal–Wallis test to test for effects of fire category (presence, absence) on stems per plot. To visualize and test for differences in size structure across plots with and without fire, we considered 10 species that are present in both fire categories. These 10 species were sorted categorically based on their bark thickness (thick or thin) and resprouting ability (cannot resprout, basal sprouters, clonal sprouters) (see Appendix S1C for details). Basal sprouters are capable of resprouting from their root collar, and clonal sprouters are capable of resprouting of producing root sprouts and root collar sprouts (Clarke et al., 2013). We used a chi‐square analysis to determine whether the fire presence influences the proportion of stems in four GBH size classes (<10 cm, 10‐20 cm, >20‐<50 cm, and >50 cm) for three functional categories of trees (basal sprouters with thin bark, basal sprouters with thick bark, and clonal resprouters with thick bark). We could not statistically consider all combinations of functional categories, as several had too few individuals in one of the treatments (e.g., species not capable of resprouting were rare in treatments with fire presence). While the sample size is inadequate for statistical analyses, the size structure of each of the 10 species is visualized in Appendix S1D.

## 3 RESULTS

**FIGURE 3:**
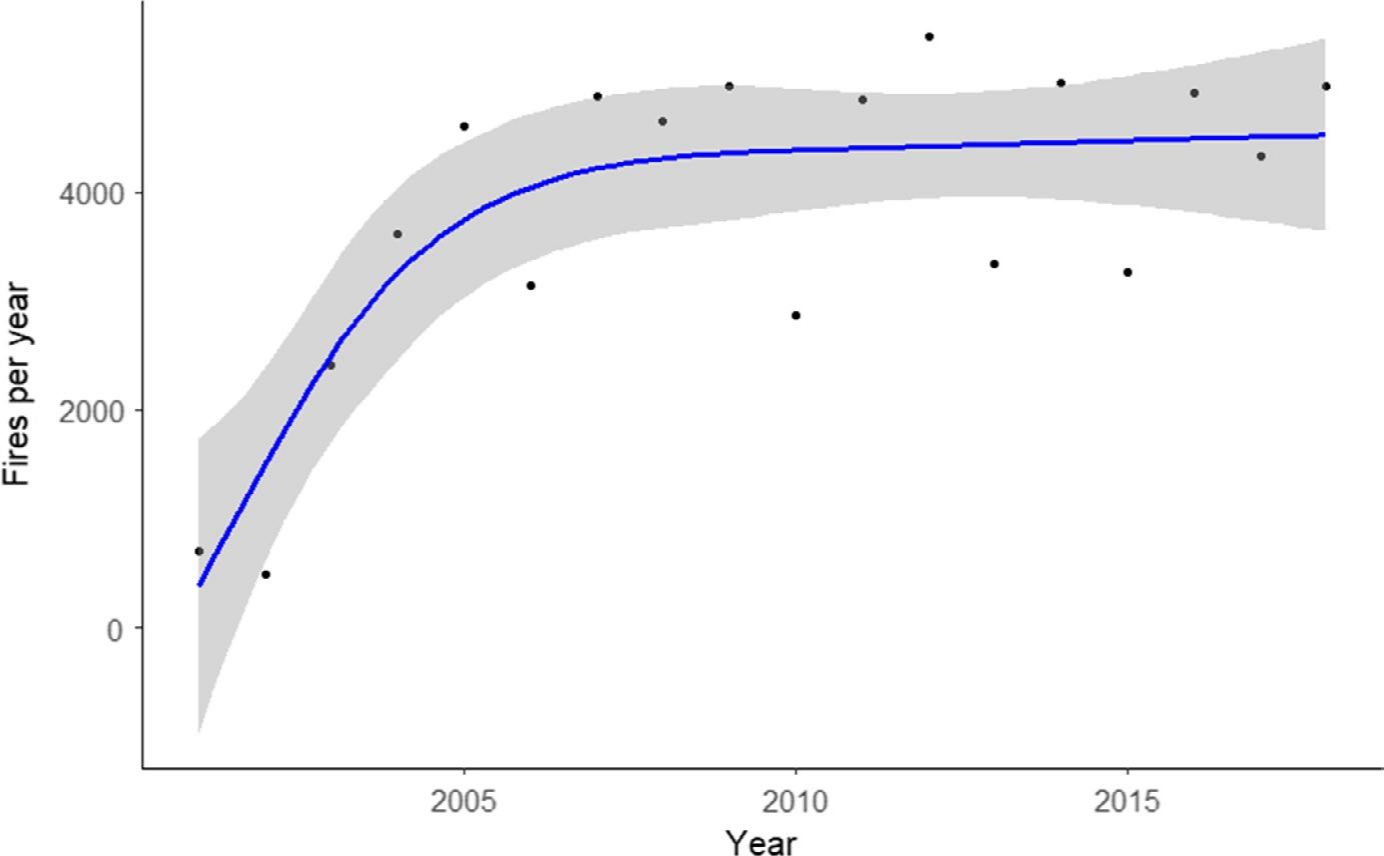
Fires per year detected from MODIS satellites from 2001 to 2018. The blue line shows the fit from a GAM with 95% confidence intervals

In our region, fires have increased in frequency from 2001 to 2018 in an asymptotic pattern (*p* < .001; Figure 3). Thus, the frequency of fire was similarly high during our two sampling years.

**FIGURE 4:**
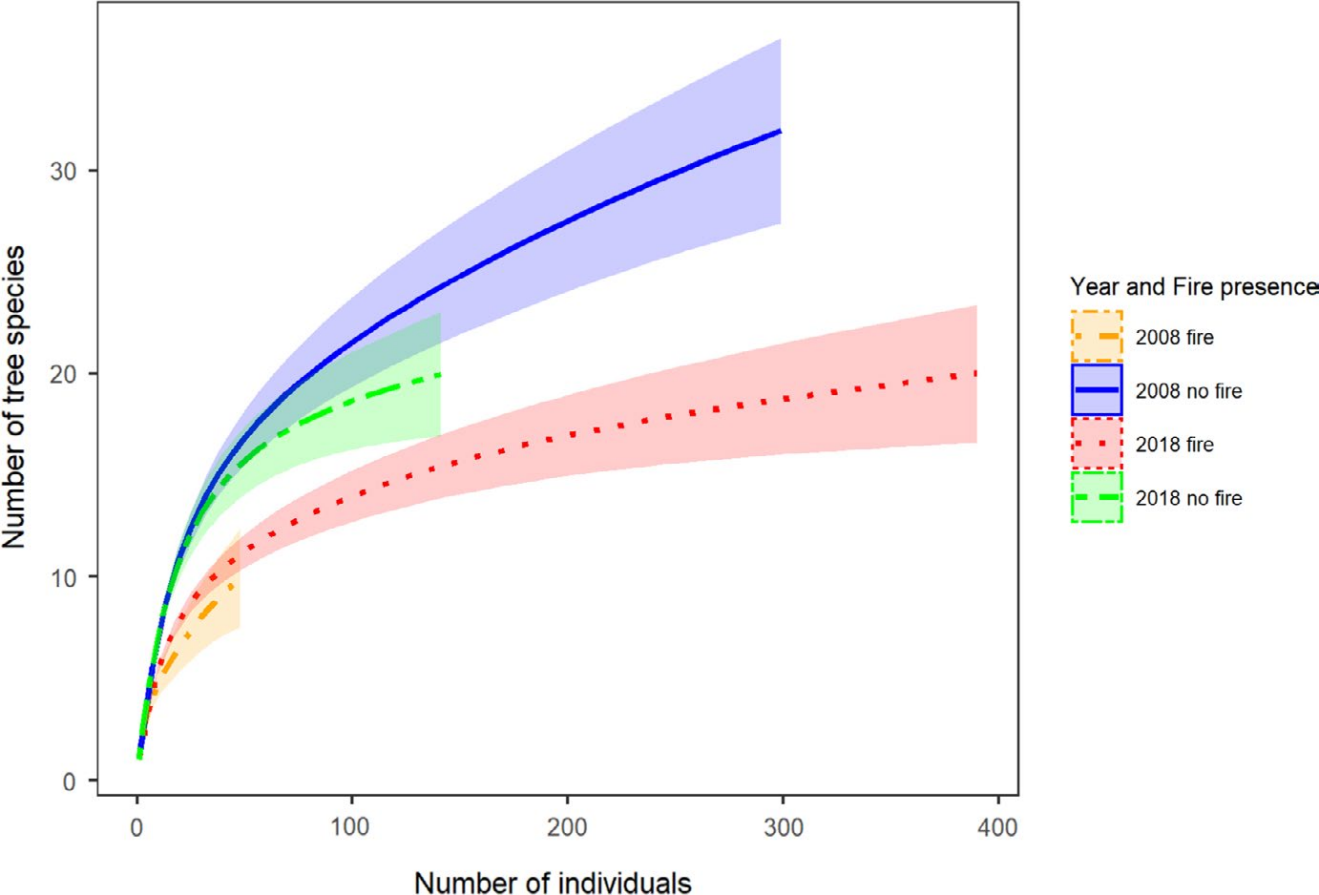
Individual‐based rarefaction curves (and 95% confidence intervals) showing tree species richness across all sample plots in four categories representing different sampling years and the presence and absence of fire

Our individual‐based rarefaction curves show that the presence of fire dramatically reduced the number of species observed in both 2008 and 2018 (Figure 4). Across plots without fire, the 95% confidence intervals of the years overlapped with each other, indicating that tree diversity remains constant across the decade of sampling in this forest reserve in the absence of fire threats. In the presence of fire, tree diversity is similarly low in both 2008 and 2018.

**FIGURE 5:**
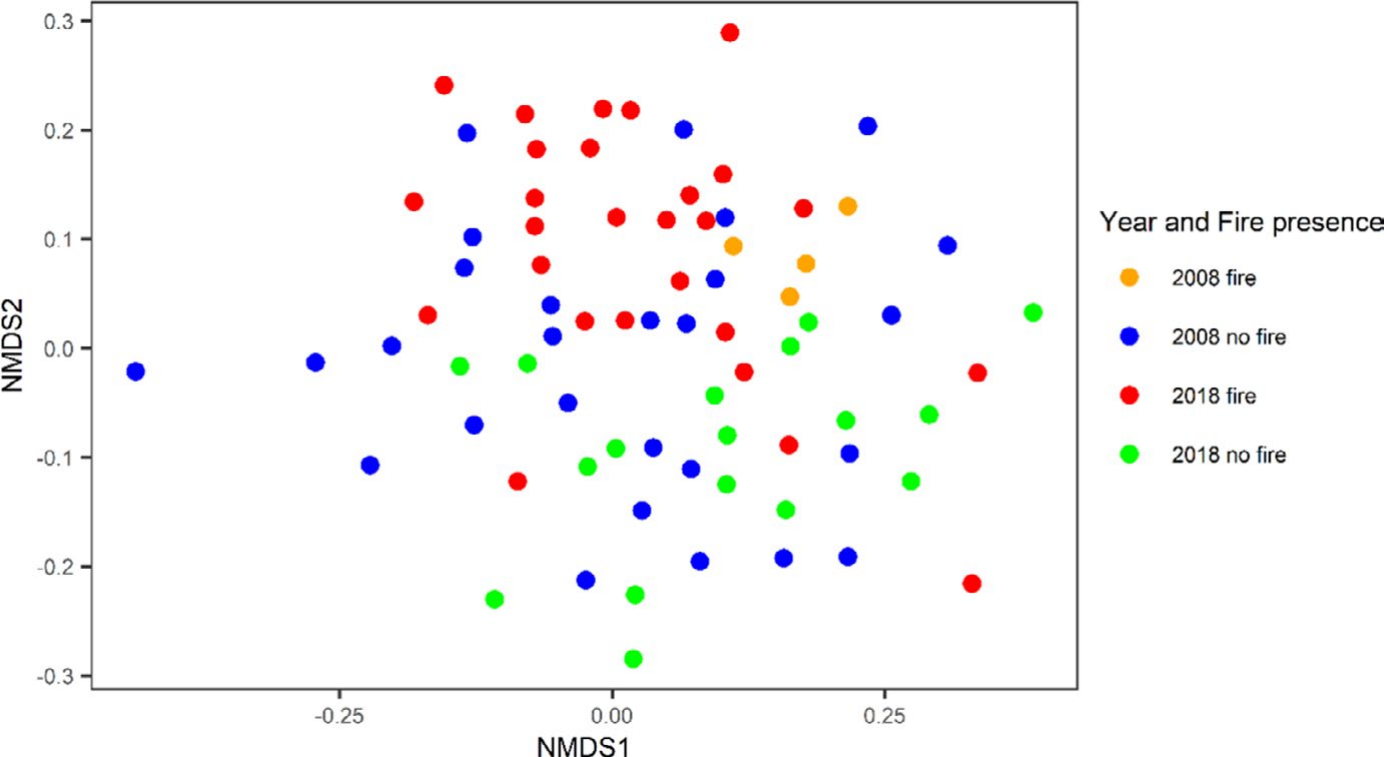
NMDS ordination of tree species composition (Bray–Curtis, stress=0.158) showing 78 sampling plots in four categories representing different sampling years and the presence and absence of fire

There was a significant effect of category (all four combinations of years and fire) on tree species composition (ANOSIM, *R* = 0.1505, *p* = .001; Figure 5) and significant pairwise differences between 2018 fire and 2018 no fire (ANOSIM, R = 0.2976, *p* = .001) and 2008 fire and 2018 fire (ANOSIM, *R* = 0.2978, *p* = .005), but no significant pairwise differences between 2008 fire and 2008 no fire (ANOSIM, *R* = 0.08436, *p* = .16) and 2008 no fire and 2018 no fire (ANOSIM, *R* = 0.06967, *p* = .053).

**FIGURE 6:**
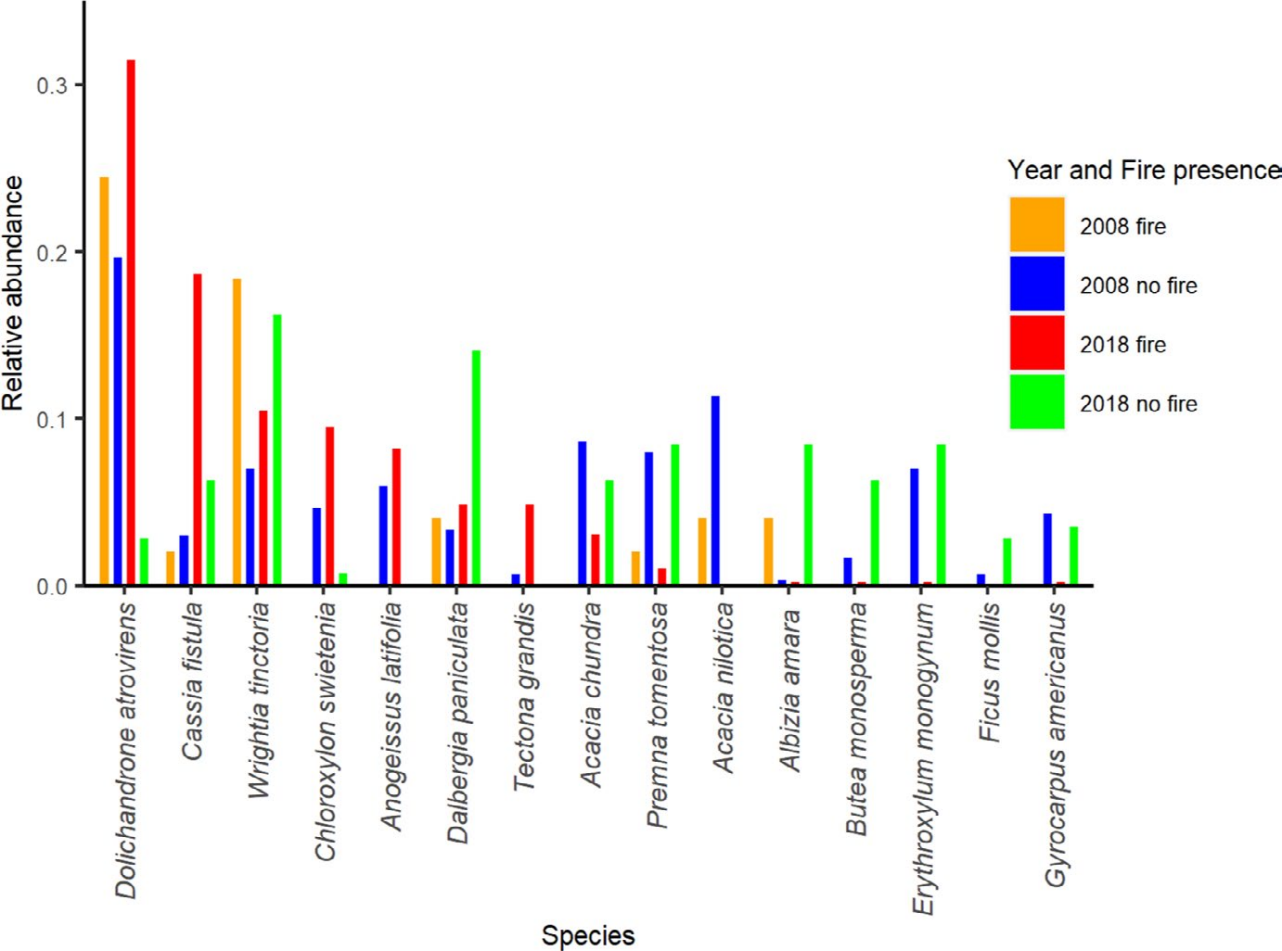
Relative abundance of tree species in four categories representing different sampling years and the presence and absence of fire. This visualization includes the 15 most common species across all sampling plots

Tree species such as *Dolichandrone atrovirens*, *Cassia fistula*, *Chloroxylon swetenia*, and *Anogeissus latifolia* are relatively common in the presence of recent fire (Figure 6). Species such as *Dalbergia paniculata*, *Premna tomentosa*, *Acacia nilotica*, *Albizia amara*, and *Erythroxylum monogynum* are relatively common in plots without recent fire incidence. Species such as *Wrightia tinctoria* are relatively common in both plots with and without recent fire incidence.

**FIGURE 7:**
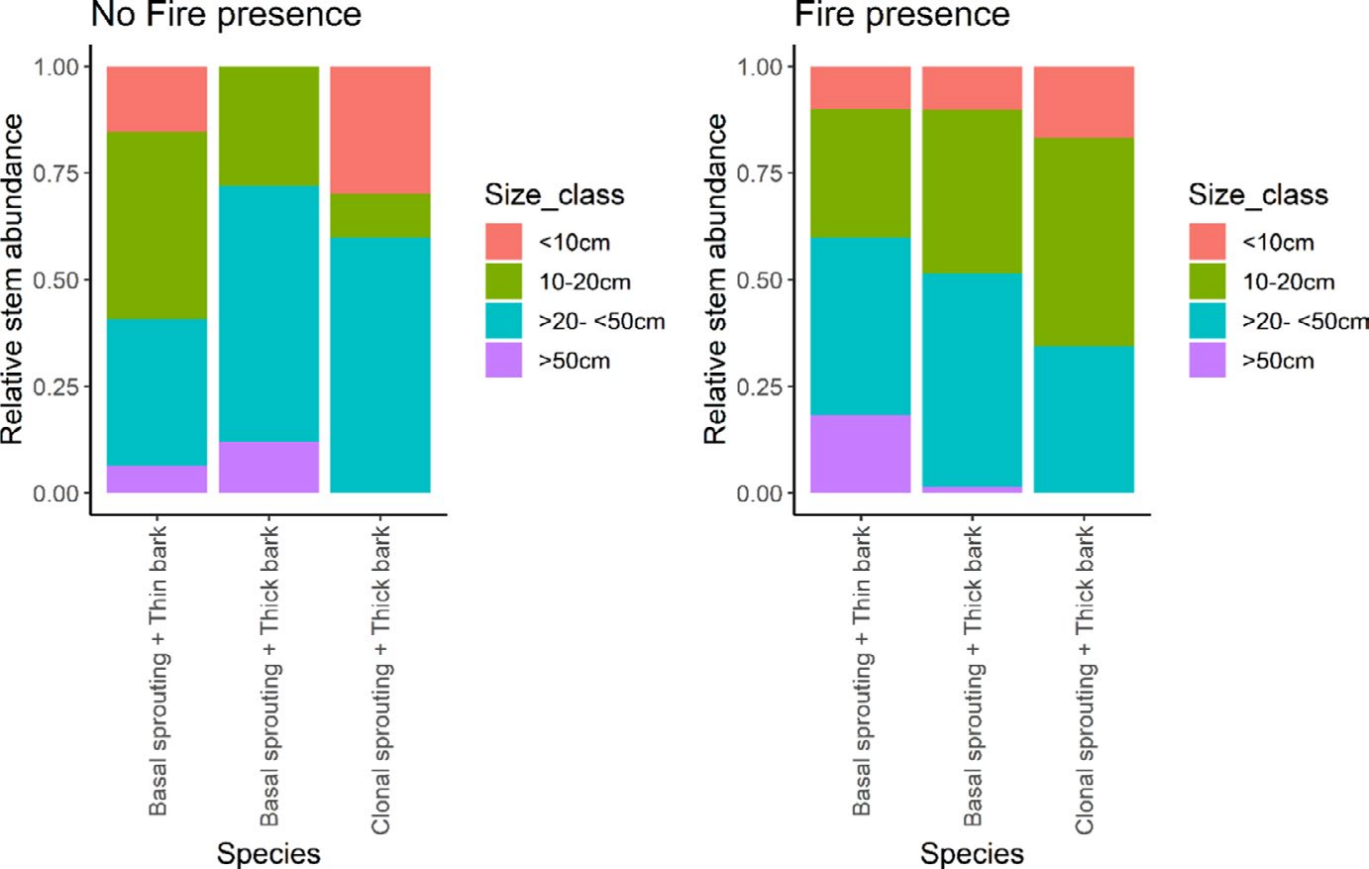
Relative number of stems for each functional group of species (considers common species in three functional group categories) that are in each size class in plots with no fire presence (left) and in plots with fire presence (right) in 2018

The density of stems was higher in plots with recent fire compared to those with no fire in 2018 (Kruskal–Wallis test, *p* = .0017). For the more common species for which we could assess size structure, there was a significant change in structure for the three categories of functional groupings. Specifically, the two most fire‐tolerant functional groups of species have relatively more individuals in the second smallest size class in plots with recent fire (10‐20 cm) (Table 1, Figure 7), whereas the least fire‐tolerant functional group (basal sprouting and thin bark) has more individuals in the two largest size classes in the presence of fire.

**TABLE 1:** Chi‐square results testing whether there are differences in size structure in the presence and absence of fire for each functional group. Individuals were pooled across species within each functional group category

Functional group and species	*p* value
Basal sprouting +Thin bark	
*Albizia amara*	.03554
*Premna tomentosa*	
*Erythroxylum monogynum*	
*Dalbergia paniculata*	
*Acacia chundra*	
Basal sprouting +Thick bark	
*Wrightia tinctoria*	.04259
Clonal sprouting +Thick bark	
*Cassia fistula*	.003327
*Chloroxylon swietenia*	
*Dolichandrone atrovirens*	

## 4 DISCUSSION

Our study finds strong effects of fire presence on tree abundance, diversity, and composition. While fire‐affected areas have greater stem density, they have lower species diversity due to increased dominance of just a few species. Similar to other studies (e.g., Saha & Howe, 2003; Sukumar et al., 2005; Verma & Jayakumar, 2015), species with fire‐tolerant traits such as thick bark and resprouting mechanisms have higher relative abundance in plots with fire. Further, fire shifts the size structure of the forest toward small resprouting individuals of species with fire‐tolerant traits.

Our decadal sampling of the Eswaramala Reserve Forest indicates rapid changes. Fires have become more frequent in the region over the past two decades. Locally, residents report that fires also occur over larger areas (FES social survey 2018 unpublished). There are reports of increased dominance of C4 grasses in the understory, especially the highly flammable *Cymbopogon coloratus,* in the forest understory (Rajendrakumar, 2014). Together, these observations suggest a grass–fire cycle, in which grasses that can tolerate fire provide fuel that can increase the frequency and/or extent of future fires (Bowman & Murphy, 2010; D’Antonio & Vitousek, 1992; Hiremath & Sundaram, 2005). In the long term, frequent fires convert forests into scrub vegetation and grasslands (Barlow & Peres, 2004; Cochrane, 2003; Kraus & Goldammer, 2007; Puyravaud et al., 1994) and cause soil erosion (Santin & Doerr, 2016). Frequently burned forests might also be more vulnerable to invasion by exotic fire‐adapted species, such as *Prosopis juliflora*, *Lantana camara*, and *Parthenium histophorus* (Hiremath & Sundaram, 2005; Saha & Hiremath, 2003). For example, *Prosopis juliflora* now occurs in our study region, but was not present 25 years ago when fires were less common (FES, 2011).

Species that have the best fire tolerance and resistance traits (i.e., capable of producing basal and root resprouts and high bark thickness), such as *Dolichandrone atrovirens, Cassia fistula* and *Chloroxylon swietenia*, dominate in the presence of fire. In particular, we noticed that *Dolichandrone atrovirens* resprouts quickly and grows fast, which could explain its high abundance, especially of individuals in the 10–20 cm size class, in recently burned plots. Species with thin bark, such as *Premna tomentosa*, *Erythroxylum monogynum,* and *Gyrocarpus americanus,* have higher relative abundance in the absence of fire and have proportionately more individuals in larger size classes in the presence of fire. *Gyrocarpus americanus* is known to not have any clonal sprouting mechanisms (Otterstorm et al., 2006), and we noticed that *Premna tomentosa*, *Erythroxylum monogynum*, and *Acacia chundra* do not resprout as quickly as species in the most fire‐tolerant functional group.

Local people cite frequent fire, along with fuelwood extraction and unregulated cutting for brickmaking, as important reasons for the reduced availability of forest resources in recent years, such as fodder, fuelwood, and nontimber forest products (FES, 2011; FES social survey, 2018 unpublished). Shifts in the abundance, size structure, and composition of trees in response to fire will affect local livelihoods and regulating ecosystem services in our region. Shepherds in this region require fodder resources for livestock that mainly comprised of sheep and goats (FES social survey, 2018 unpublished). After a fire, if there is also precipitation due to summer rains, resprouting C4 grasses are palatable for a short time period and provide fodder resources to grazing animals in the dry season (FES social survey 2018 unpublished). Preferred fodder species for goats are *Dolichandrone atrovirens, Acacia chundra, Albizia amara, Azadirachta indica, Hardwickia binata,* and *Premna tomentosa*. Of these, only *Dolichandrone atrovirens* is common and abundant in the presence of fire. Sheep prefer *Azadirachta indica* and *Hardwickia binata*, which are already rare species at our site. Further, several species that provide valuable timber and nontimber forest products (i.e., ethnomedical uses) are rare in all plots or are particularly rare in plots with recent fire, such as *Dalbergia paniculata*, *Premna tomentosa*, *Gyrocarpus americanus*, *Erythroxylum monogynum*, *Ficus mollis*, and *Butea monosperma* (FES, 2011; FES social survey, 2018 unpublished).

A multistakeholder approach is already underway in this region to activate broad‐base, community‐led conservation action. The Foundation for Ecological Security (FES) is a local conservation NGO that is promoting protection of resources by collaborating with forest‐dependent communities. FES engages in strengthening democratic village institutions for collective governance through helping communities secure tenure over them and undertake measures for the restoration of forest resources. FES has been engaging the rural community members to understand the effects of fire through campaigns and exercises on taking precautions to prevent forest fires and also on the measures of dousing fire.

We suggest that future research should experimentally exclude fire and follow permanently tagged individuals and plots, to allow opportunities to quantify demographic vital rates and project future population and community dynamics. While we believe the short‐term results from our observational study would match those of an experimental study, as the spatial locations of burned versus. unburned sites are more likely to be random than due to differences in their physical and biological characters, it would be useful to have an experimental system that was designed for the long‐term exclusion of fire. Further, while the presence of small, resprouting individuals of fire‐tolerant species such as *Dolichandrone atrovirens* can maintain the size of a population in the intermediate future, recruitment is necessary for long‐term viability (Bond & Midgley, 2001; Clark et al., 2013). Compared with nonsprouters, resprouters allocate more biomass to roots, have lower seed output and seedling establishment, and take longer to reach a reproductive stage (Bond & Midgley, 2001; Clark et al., 2013). Thus, by favoring resprouting species, fire changes the functional composition of forests.

The dry forests in the Deccan Peninsula and Eastern Ghats face a serious threat from climate change (Das & Behera, 2019; Remya et al., 2015), which may extend the length of the dry season. These changes could directly cause the loss of species in our system, which cannot tolerate these new conditions. Currently, there is a lack of species distribution models for species in the Eastern Ghats, and this is an important topic for future research. Further, climate could also extend the fire season, and/or change the frequency, extent, or intensity of fires (Kale et al., 2017). Currently, the long dry season makes it difficult for many tree species to recover from fire. The interactive threats of fire and climate change could cause the local extinction of species that might otherwise be present in the presence of either threat.

## 5 CONCLUSIONS

In our region, fire has increased in frequency across the past two decades. Fire poses an urgent threat to forests, as it results in the dominance of a few, fire‐tolerant tree species, and a shift in the size structure toward smaller, resprouting individuals. These results demonstrate that conservation actions are needed to prevent further degradation of forests in this region that support local livelihood and other valuable ecosystem services.

## CONFLICT OF INTEREST

The authors declare that they have no conflict of interest.

## AUTHOR CONTRIBUTION

**Neeraja U.V:** Data curation (equal); Formal analysis (lead); Investigation (lead); Visualization (lead); Writing‐original draft (lead); Writing‐review & editing (lead). **S Rajendrakumar:** Data curation (equal); Investigation (equal); Methodology (supporting); Writing‐review & editing (equal). **CS Saneesh:** Investigation (equal); Writing‐review & editing (equal). **Venkat Dyda:** Funding acquisition (supporting); Supervision (supporting); Writing‐review & editing (equal). **Tiffany M Knight:** Conceptualization (lead); Formal analysis (supporting); Funding acquisition (lead); Project administration (lead); Resources (lead); Supervision (lead); Visualization (supporting); Writing‐original draft (supporting); Writing‐review & editing (equal).

## AUTHOR CONTRIBUTIONS

U.V. Neeraja: Data curation (equal); formal analysis (lead); investigation (lead); visualization (lead); writing—original draft (lead); writing—review and editing (equal). S. Rajendrakumar: Data curation (equal); investigation (equal); writing—review and editing (equal). C.S. Saneesh: Investigation (equal); writing—review and editing (equal). Venkat Dyda: Supervision (supporting); writing—review and editing (equal); funding acquisition (supporting). Tiffany M. Knight: Conceptualization (lead); formal analysis (supporting); funding acquisition (lead); project administration (lead); resources (lead); supervision (lead); visualization (supporting); writing—original draft (supporting); writing—review and editing (equal).

## Supporting information

Supplementary Material

## Data Availability

Tree species abundance data for both sampling years and size structure data for 2018 are available on Dryad https://doi.org/10.5061/dryad.p2ngf1vq8
