# Supplementary material for: Fire alters diversity, composition, and structure of dry tropical forests in the Eastern Ghats
Source: Ecol Evol. 2021 May 1;11(11):6593–603. doi: 10.1002/ece3.7514 (PMC8207398; doi:10.1002/ece3.7514)
Supplement: Supplementary file 1 — Supplementary Material [file ECE3-11-6593-s001.docx]

**Appendices**

Appendix A. Community dissimilarity and geographic distance of sampling plots.

We used Mantel Tests with the *mantel* function from the package *vegan* in R to test the relationship between geographic distance and pairwise dissimilarity (Bray Curtis index) in 2008 and 2018. We used the *vegdist* function to compute dissimilarity indices for community composition data from package *vegan* in R. Geographic distance between sites was calculated as Haversine distance from the package *geosphere* (Hijmans, 2016) in R. All statistical analyses were conducted in R programming version 3.6.2 (R Core Team 2017).


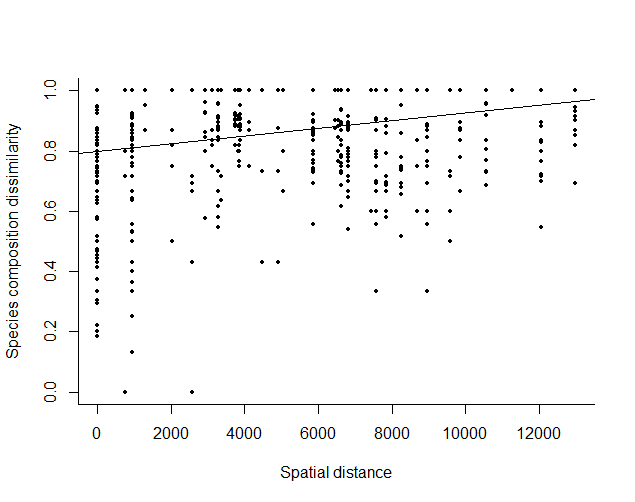


Fig. A.1. Relationship between community dissimilarity and geographic distance for 2008 plots. Pairwise dissimilarities are plotted as a function of the distance between the sampling plots. Mantel statistic r: 0.2363, p<0.001


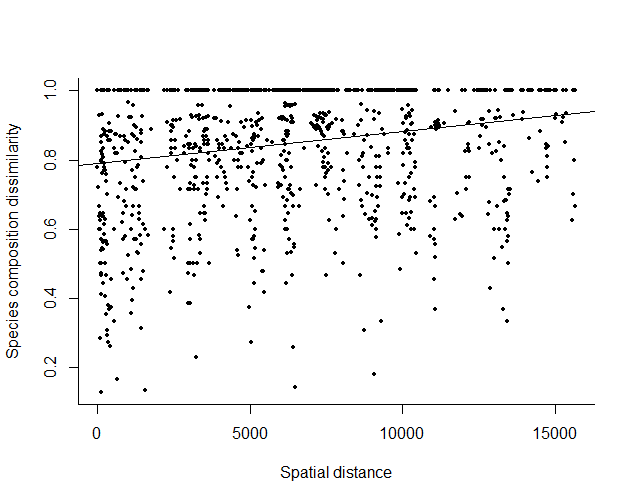


Fig. A.2. Relationship between community dissimilarity and geographic distance for 2018 plots. Pairwise dissimilarities are plotted as a function of the distance between the sampling plots. Mantel statistic r: 0.1999, p<0.001

Appendix B. Tree species composition across four categories representing sampling years and the presence and absence of fire. This visualization includes eight plots that had only one tree species. Because two of these plots are clear outliers, these eight plots were removed from the statistical analysis and visualization in the main paper.


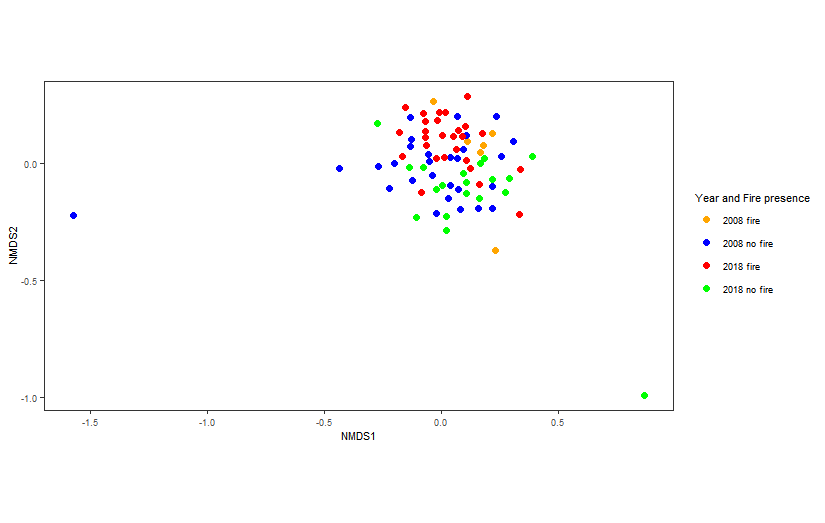


Fig. B.1 NMDS ordination of tree species composition (stress=0.158) showing all the vegetation plots in four categories representing sampling years and the presence and absence of fire.

Appendix C. Categories of bark thickness and resprouting ability for 10 focal tree species.

| **Family** | **Species** | **Mean GBH** | **Mean bark thickness** | **Relative bark thickness** | **Category** |
| --- | --- | --- | --- | --- | --- |
| Fabaceae | *Albizia amara* | 60.26 | 0.26 | 1.35 | Thin (Anil and Parthasarthy 2016) |
| Erythroxylaceae | *Erythroxylum monogynum* | |  |  | Thin (Sasidharan 2006) |
| Fabaceae | *Dalbergia paniculata* | 52.40 | 0.610 | 3.65 | Thin |
| Hernandiaceae | *Gyrocarpus americanus* | 43.5 | 1 | 7.22 | Thin |
| Fabaceae | *Acacia chundra* | 40.69 | 0.98 | 7.57 | Thin |
| Lamiaceae | *Premna tomentosa* | 21 | 0.53 | 7.97 | Thin |
| Bignoniaceae | *Dolichandrone atrovirens* | 36.12 | 1.03 | 8.99 | Thick |
| Fabaceae | *Cassia fistula* | 26.25 | 0.80 | 9.62 | Thick |
| Rutaceae | *Chloroxylon swietenia* | 37.6 | 1.18 | 9.85 | Thick |
| Apocynaceae | *Wrightia tinctoria* | 28.51 | 0.92 | 10.16 | Thick |

Table C.1. Categories of bark thickness for 10 focal tree species. To quantify bark thickness, co-author Saneesh CS measured girth at breast height (GBH) and bark thickness measurements of trees. Relative bark thickness was calculated as (Mean Bark thickness/ Mean diameter)*100 following Hoffman et al. 2012, Lawes et al. 2013. Tree species were grouped into thin and thick bark based on quantile grouping using *cut2* function from *Hmisc* package in R to make the continuous numerical variable into categorical. Additional citations supporting the categories of thin or thick for each focal tree species are also given in the Category column

| **Family** | **Species** | **Resprouting type** |
| --- | --- | --- |
| Fabaceae | *Albizia amara* | Basal (own observation) |
| Erythroxylaceae | *Erythroxylum monogynum* | Basal (own observation) |
| Fabaceae | *Dalbergia paniculata* | Basal (Saha and Howe 2003) |
| Hernandiaceae | *Gyrocarpus americanus* | Non sprouting (Otterstrom 2006) |
| Fabaceae | *Acacia chundra* | Basal (own observation) |
| Lamiaceae | *Premna tomentosa* | Basal (own observation) |
| Bignoniaceae | *Dolichandrone atrovirens* | Clonal (own observation) |
| Fabaceae | *Cassia fistula* | Clonal (own observation) |
| Rutaceae | *Chloroxylon swietenia* | Clonal (own observation) |
| Apocynaceae | *Wrightia tinctoria* | Basal (own observation) |

Table C.2. Categories of resprouting type for 10 focal tree species. Information on resprouting is based on field observations of the authors and/or the literature cited. Basal sprouters are capable of resprouting from their root collar. Clonal sprouters are capable of producing root sprouts and root collar sprouts

Appendix D: Stems density (stems per plot) in plots with no fire presence and fire presence.


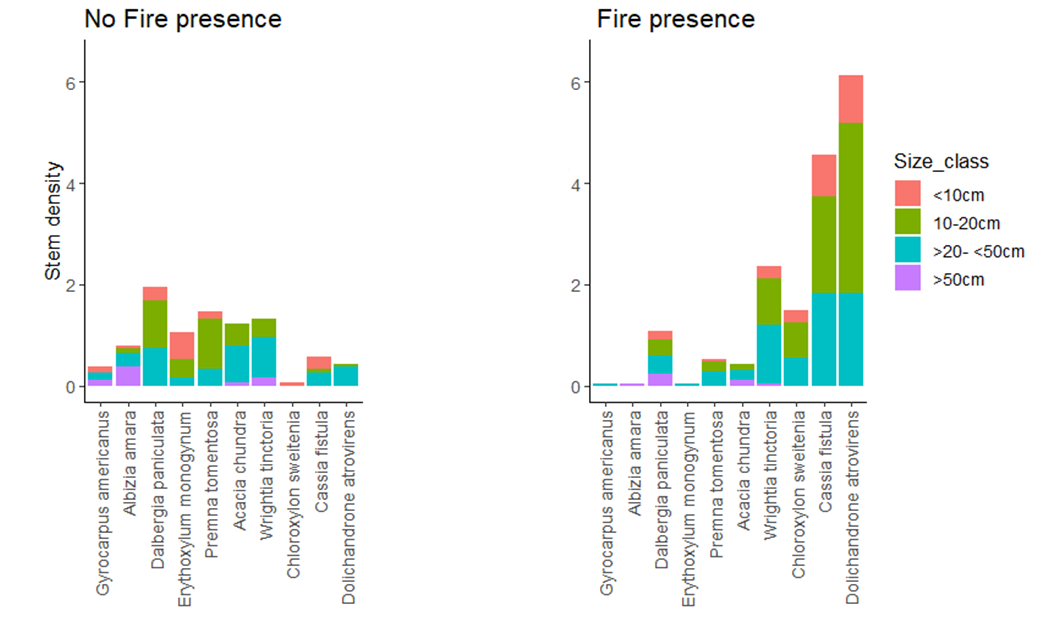


Fig. D. 1 Stems per plot of 10 focal tree species in plots with no fire presence (left) and plots with fire presence (right) in 2018.

**References.**

Hijmans, Robert J., 2016. Geosphere: Spherical Trigonometry. https://CRAN. R-project. org/package= geosphere

Hedge V, Chandran MDS & Gadgil M., 1998. Variation in Bark Thickness in a Tropical Forest Community of Western Ghats in India; Functional Ecology 12: 313-318. <https://doi.org/10.1046/j.1365-2435.1998.00191.x>

Saha, S., Howe, H.F., 2003. Species composition and fire in a dry deciduous forest. Ecology 84 (12), 3118–3123. https://doi.org/10.1890/02-3051

Otterstrom, S. M., Schwartz, M. W., Velazquez-Rocha, I., 2006. Responses to Fire in selected tropical dry forest trees. Biotropica 38: 592–598. https:/doi.org/ 10.1111/j.1744-7429.2006.00188.x.

Anil, K., Parthasarathy, N., 2016. Bark traits of woody species and bark resource use by faunal community in tropical dry evergreen forest of India. Int. J. Curr. Res. Biosci. Plant Biol. 3(2), 77-90. http://dx.doi.org/10.20546/ijcrbp.2016.302.010

Sashidharan N, 2006. Illustrated Manual on Tree Flora of Kerala supplemented with computer aided identification. Kerala Forest Research Institute Research report 282.

Hoffmann, W.A., E.L. Geiger, S.G. Gotsch, D.R. Rossatto, L.C.R. Silva, O.L. Lau, M. Haridasan, and A.C. Franco. 2012. Ecological thresholds at the savanna-forest boundary: how plant traits, resources and fire govern the distribution of tropical biomes.Ecology Letters 15: 759–768. doi: [10.1111/j.1461-0248.2012.01789.x](https://doi.org/10.1111/j.1461-0248.2012.01789.x)

Lawes MJ, Midgley JJ, Clarke PJ. 2013. Costs and benefits of relative bark thickness in relation to fire damage: A savanna/forest contrast. Journal of Ecology. 101. doi: 10.1111/1365-2745.12035.
